# Supplementary material for: The Quality and Bacterial Community Changes in Freshwater Crawfish Stored at 4 °C in Vacuum Packaging
Source: Molecules. 2022 Dec 6;27(23):8618. doi: 10.3390/molecules27238618 (PMC9740484; doi:10.3390/molecules27238618)
Supplement: Supplementary file 1 [file molecules-27-08618-s001.zip › molecules-1994477-supplementary.pdf]

Table S1 Observed valid sequence and OUT numbers in different samples

sample	Total reads	Identified reads	OUTs
C-0-D_0	14892	14466	193
C-0-D_{12}	19965	19802	41

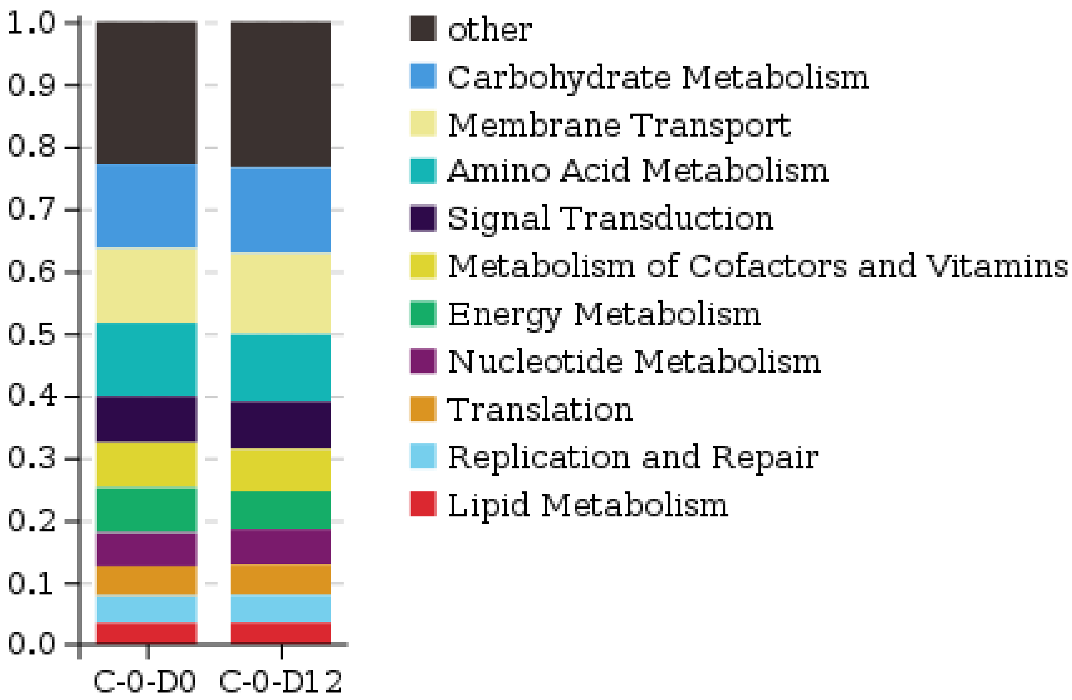

Figure.S1 Function stacking map

Table S2 Techniques in monitoring spoilage microbiota

**Techniques**	**Crustaceans**	**Bacteria**	**Level**	**Alpha diversity**	**References**
**16s rRNA high-thought Next-generation sequencing**	*Procambarus clarkia*	*Aeromonas, Macrococcus, Vibrio sp., Acinetobacter, Citrobacter, Enterobacter*	Genus	Shannon index 2.5<br>Simpson index 0.148	[52]
	Procambarus clarkii	*Shewanella sp., Carnobacterium sp., Brochothrix sp., Psychrobacter sp., Vagococcus sp., Acinetobacter sp.*	Genus	Shannon index 6.58<br>Simpson index 0.97	[53]
	Litopenaeus vannamei	*Vibrio, Candidatus, Bacilloplama, Photobacterium, Candidatus, Bacilloplama*	Genus		[54]
	Penaeus vannamei	*Proteobacteria Sphingomonas, Carnobacterium, Psychrobacter, Psychro-bacter, Pseudomonas, Shewanella, Acinetobacter, Vibrio*	Genus		[55]
	Litopenaeus vannamei	*Pseudoalteromonas, Aliivibrio, Photobacterium*	Genus		[56]
	Parapenaeus Longirostris	*Psychrobacter,Brevundimonas,Stenotrophomo nas, Staphylococcus,Legionella,Acinetobacter, Bacillus, Escherichia-Shigella, Enterococcus, Enterobacter, Klebsiella*	Genus		[57]
	Solenocera melantho	*Vagococcus, Shewanella, Pseudomonas, Psychrobacter, Trichococcus*	Genus		[58]
	Pacific White Shrimp	*Acinetobacter, Psychrobacter, Shewanella, Carnobacterium,Pseudomonas, Vibrio*	Genus		[59]
	Ctenopharyngodon idellus	*Pseudomonas, Aeromonas, Shewanella*	Genus		[60]
**Culture dependent 16S rRNA sequencing**	*Procambarus clarkii*	*Aeromonas jandaei*	Species		[61]
	Litopenaeus Vannamei	*lactic acid bacteria, Pseu-domonas spp., Shewanella spp., Aeromonas spp.*	Genus		[62]

	Litopenaeus Vannamei	*Enterobacter, Acinetobacter, Pseudomonas,Aeromonas, Aeromonas, Enterococcus*	Genus		[63]
	Penaeus notialis	*lactic acid bacteria, Enterobacteriaceae, Pseudomonas spp.*	Genus		[64]
**Culture dependent API kits**	*Fenneropenaeus indicus*	*Actinomyces, Sphingobacterium multivorum, Vibrio proteolyticus, Staphylococcus cohnii, S. auricularis*	Species	Simpson index 0.9	[65]
**DGGE**	*Exopalaemon Modemus*	*Pseudomonas sp., Shewanella, Flavobacterium, Staphylococcus*<br>*lactic acid bacteria, Pseudomonas sp., Shewanella, Flavobacterium*	Genus		[66]
	Crangon crangon	*Psychrobacter immobilis, Psychrobacter cibarius. Pseudoalteromonas nigrifaciens, Pseudoalteromonas elyakovii, Pseudoalteromonas paragorgicola Planocuccus, Exiguobacterium, Carnobacterium,Pseudomonas, Chryseobacterium Staphylococcus*	Species		[67]
**PMA combined with DGGE**	*Litopenaeus vannamei*	*Acinetobacter sp., Vibrio sp., Aeromonas sp., Lactococcus sp., Exiguobacterium sp., Kurthia sp..*	Genus	Shannon index nearly 2.8	[60]
**TTGE**	*Litopenaeus vannamei*	*C. maltaromaticum and S. baltica.*	Species		[61]
